# Comparison of 24-Hour Recall and 3-Day Food Records during the Complementary Feeding Period in Thai Infants and Evaluation of Plasma Amino Acids as Markers of Protein Intake

**DOI:** 10.3390/nu13020653

**Published:** 2021-02-17

**Authors:** Kulnipa Kittisakmontri, Julie Lanigan, Areeporn Sangcakul, Thipwimol Tim-Aroon, Pornchai Meemaew, Kanticha Wangaueattachon, Mary Fewtrell

**Affiliations:** 1 Childhood Nutrition Research Centre, University College London Great Ormand Street Institute of Child Health, London WC1N 1EH, UK; j.lanigan@ucl.ac.uk (J.L.); m.fewtrell@ucl.ac.uk (M.F.); 2 Division of Paediatric nutrition, Department of Paediatrics, Faculty of Medicine, Chiang Mai University, Chiang Mai 50200, Thailand; 3 Division of Clinical Chemistry, Department of Pathology, Faculty of Medicine Ramathibodi Hospital, Mahidol University, Bangkok 10400, Thailand; areeporn.cho@mahidol.ac.th (A.S.); pornchai.mee@mahidol.ac.th (P.M.); kanticha.wan@mahidol.ac.th (K.W.); 4 Division of Medical Genetics, Department of Paediatrics, Faculty of Medicine Ramathibodi Hospital, Mahidol University, Bangkok 10400, Thailand; Thipwimol.tim@mahidol.ac.th

**Keywords:** validity, dietary assessment tool, protein intake, plasma amino acids, protein biomarker, infant nutrition, complementary feeding, lower-middle income countries

## Abstract

Background: An accurate and reliable measurement of nutrient intake is the first and foremost step in order to optimise infant nutrition and evaluate its impact on health outcomes. However, research on the validity of dietary assessment tools used during the weaning period is limited, especially in lower-middle income countries. The primary aim of this study was to evaluate relative validity of a 24-h recall method (24-HR) using a 3-day food record (3-DFR). A secondary aim was to investigate association between protein intake from 3-DFR and plasma amino acids as a potential protein biomarker. Methods A multicentre, prospective cohort study was conducted in Chiang Mai, Thailand from June 2018 to May 2019. Food consumption data were collected in healthy infants using 24-HR and 3-DFR at 9 and 12 months of age. Blood samples were obtained at 12 months (M). Plasma amino acids were analysed using high performance liquid chromatography. Results Of 145 infants, 49% were female. At group level, paired *t*-tests/Wilcoxon signed rank tests did not show significant differences between average nutrient intakes from the 2 dietary assessment methods, except for vitamin A and vitamin C. Weighted kappa (Kw) was acceptable for all nutrients, except for vitamin A intake at 9 M (Kw = 0.15). The Bland–Altman analyses were unbiased for most nutrients with variable limits of agreement. At individual level, correlation coefficients (r) ranged from acceptable to excellent (r = 0.37–0.87) while cross-classifications showed acceptable outcomes, except for vitamin A. Multivariate analyses showed significant associations between protein intake at 12 M from the 3-DFR and plasma concentrations of branched-chain amino acids (BCAA) and essential amino acids (EAA), even after adjusting for gender, milk feeding type and energy intake. Conclusions For infants aged 9–12 M, a 24-HR can be used as a more practical alternative to a 3-DFR for most nutrients although caution is required for some micronutrients, especially vitamin A. A repeated interview might further improve the accuracy. Furthermore, protein intake, particularly animal-based protein, significantly predicted plasma BCAA and EAA concentrations regardless of gender, type of milk feeding and energy consumption.

## 1. Introduction

The double burden of malnutrition (DBM) is an emerging problem affecting global populations especially in lower-middle income countries (LMICs) [1]. It can begin very early in life and continues its impact throughout the life-course [2]. Understanding how early-life nutrition influences child health and nutritional status is a fundamental step to prevent and manage the DBM. However, accurate and reliable estimates of dietary intake are needed to draw conclusions or develop pragmatic guidelines from nutritional studies. These should be population specific and based on data obtained with validated tools.

Despite the considerable effort invested to develop novel dietary assessment tools based on current technologies, the multiple-day weighed food record is still widely accepted as the best reference method to assess the relative validity of other dietary assessment methods [3]. However, this technique is quite expensive and has a high respondent burden which often leads to low participation and high attrition rates [4]. This is particularly the case for infants and young children living in LMICs where respondents may live in challenging conditions and have limited time or resources available for measuring foods or describing recipes in detail. Therefore, most nutritional studies related to infant and young child feeding in LMICs collect dietary data using tools that place less burden on participants. Several studies found that food frequency questionnaires (FFQ), dietary recalls and estimated food diaries also provide accurate and reliable dietary assessments in infants and young children [5,6,7,8,9,10,11,12,13,14,15,16]. However, given the small numbers of validated tools, use of different types of household utensils and locally produced foods, more validation studies from LMICs are needed.

As dietary assessment methods are unable to measure true nutrient intake and are mainly based on subjective information, biomarkers may be more accurate and provide an objective measurement of nutrients. However, few studies in the field of infant and child nutrition have validated their dietary assessment tools with biomarkers, for reasons that are well-described [4,17,18]. The most common biomarkers used to validate dietary intake in these populations are the doubly labelled water technique to evaluate energy consumption and fatty acid analysis to assess fatty acids intake from foods [3,11,19,20]. The standard biomarker method for assessment of protein intake is the 24-h urine nitrogen analysis. However, this has never been used to validate protein intakes of infant and child populations due to the practical difficulties involved. By contrast, metabolomic analyses are less intensive, require fewer samples, offer an alternative method to evaluate protein intake and could be linked to health conditions or diseases. Plasma amino acid profiles may be promising biomarkers [17,21,22]. Dietary protein is the only source of essential amino acids in humans, although different protein sources have different quality. Animal source food is known as a superior source of protein compared to plant-based foods and some studies show that omnivorous adults have different plasma amino acids compared with vegetarians and vegans [23]. In addition, a multicentre, randomised controlled trial in 5 European countries demonstrated that high protein intake from infant formula increased plasma branched-chain amino acids (sum of plasma Leucine, Isoleucine and Valine) in infants aged 6 months [24]. However, studies in adults that investigated associations between dietary protein and plasma amino acids are inconclusive [25] and there is a lack of studies in infants and children.

The present study aimed to (1) investigate the relative validity of a 24-h dietary recall (24-HR) compared with an estimated 3-day food record (3-DFR) in Thai infants representing a LMIC population; and (2) to evaluate associations between protein intake from the 3-DFR and plasma amino acids as a potential protein biomarker.

## 2. Materials and Methods

### 2.1. Study Design and Participants

A multicentre, prospective cohort study was conducted in northern Thailand between June 2018 and May 2019. Eligible participants were healthy-term infants who attended the well-baby clinics of three hospitals located in Chiang Mai province for immunization and health surveillance. Sample size was calculated to detect a 0.5 standard deviation difference in anthropometric measurements between infants who received red meat more often or less frequently using 80% power and 0.05 significance level. The total number of participants from the calculation was 126. Full details of the study protocol have been published previously [26]. At 9 and 12 months (M) of age, participants’ caregivers were asked to record their infant’s daily consumption for 3 days within the same week, whether consecutive or non-consecutive, in the 3-DFR. The 24-HR records were obtained in interviews conducted by a single paediatric nutrition-trained paediatrician at the well-baby clinics. Blood samples were collected at 12 M by healthcare professionals at those clinics.

### 2.2. Dietary Assessment Tools

Before caregivers were asked to record the 3-DFR, all families attended a brief session by the same paediatric nutrition-trained paediatrician to demonstrate how to estimate food intake using household utensils and record the 3-DFR appropriately. The 3-DFR contains four main parts. The first part shows two-dimensional pictures of various types of spoons with labels. The second part includes some examples of dietary records and is followed by the recording section. This section has free spaces for caregivers to complete the date, mealtimes, and details of food consumption. The recipe section is the last part, where caregivers can freely describe the recipes recorded in the third part if they are used in multiple meals or contain many ingredients. For the 24-HR, the paediatrician interviewed caregivers when they brought their children to the well-baby clinics at 9 and 12 M. The information included in the 24-HR was date of record, mealtime, and amount of food consumed. After the interview, the paediatrician checked for any unclear information in the 3-DFR that the caregivers had returned and resolved the issue. If the 3-DFR had not been completed, the caregivers were asked to return it to the researcher later or send it by post. Breastfeeding was recorded as duration of feeding (minutes per feeding). Conversion from breastfeeding duration to amount of breast milk intake was calculated following two techniques from Lanigan et al. [11] and Olaya et al. [27]. The average intake from the two techniques was used in the analyses.

### 2.3. Food Composition Programme

The study mainly used the Thai food composition programme called INMUCAL-Nutrients, version 4.0 (2018), developed by the Institute of Nutrition, Mahidol University, Thailand, to convert dietary data to nutrient intakes [28]. Micronutrients including calcium (Ca), phosphorous (P), iron (Fe), zinc (Zn), vitamin A, vitamin B1, vitamin B2 and vitamin C were reported along with energy and macronutrients. In some cases when nutrient profiles were not available in this programme, other reliable sources such as the United States Department of Agriculture (USDA) or the Food and Agriculture Organization of the United Nations (FAO) were used instead. For commercial products, the nutritional information for the specific products were obtained if they were not included in the INMUCAL-Nutrients programme.

### 2.4. Plasma Amino Acids Analysis

Sub-group analysis was performed using selected blood samples for measuring plasma amino acids concentrations. The aim was to compare plasma levels of those infants consuming protein in the highest and lowest quartile for each protein source, namely dairy including breast milk, non-dairy animal-based and plant-based protein. According to Lanigan et al., 4 days of food records should be considered in order to provide an accurate measurement of protein intake of infants and toddlers [29] thus data from the 3-DFR were used to select blood samples. Daily protein–energy ratio (%PE) from each protein source at 12 M was classified into quartiles and presented as the lowest (first quartile), median (second to third quartile) and highest intake (forth quartile). For the comparison of each of the protein sources, the included infants had to have median intakes of the other protein sources. Fifty-four plasma samples were selected for these analyses.

Non-fasting venous blood was collected from infants aged 12 M into EDTA tubes and centrifuged at 4 °C to prepare the plasma on the same day. Plasma samples were aliquoted into 1 mL and stored in a −20 °C freezer until analysis. Samples from fifty-four infants were selected for plasma amino acids analysis. The plasma samples were initially deproteinized with 6% sulfosalicylic acid (1:1 *v*/*v*). After mixing, the samples were centrifuged at 10,000 rpm for 10 min. Then 80 μL of the supernatant from each sample was added to 20 μL of Norleucine as an internal standard and the pH adjusted to 2.2 with lithium hydroxide before analysis according to the method of Shapira et al. [30]. Free amino acids were determined by ion exchange chromatography using the Biochrome 30+ automatic amino acid analyser (Biochrome Ltd., Cambridge, United Kingdom). A series of lithium buffer solutions were run through a lithium column containing the amino acids in solution. Individual amino acids were eluted according to their pH. Post-column derivatization with ninhydrin was utilized to elicit a spectrum of colours at different wavelengths (440 and 570 nm). Data analysis was performed using the software EZChrome Elite (SIM GmbH, Germany). Repeatability and reproducibility were ±1.5% and within ±5%, respectively. The sensitivity was shown as a detection of 15 ρmole at signal/noise ratio of 3:1. The quality control for this analysis was regularly checked with both internal and external standards.

### 2.5. Statistical Analysis

Relative validation of the 24-HR using the 3-DFR as the reference method was performed at both group and individual level. At the group level, statistical tests including Paired *t*-test/Wilcoxon signed rank test, percent mean difference, weighted kappa (Kw) and Bland–Altman plots. Spearman’s correlation and cross-classification (quartiles) were calculated representing validity at an individual level. The results from the statistical tests were interpreted by using suggested values from Lombard et al. [31]. Plasma amino acids were presented as the sums of the concentrations of (1) branched chain amino acids (BCAA: leucine, isoleucine and valine), (2) essential amino acids (EAA: leucine, isoleucine, valine, methionine, threonine, phenylalanine, tryptophan, lysine and histidine), (3) non-essential amino acids (NEAA: glycine, alanine, proline, serine, cysteine, aspartate, glutamate, asparagine, glutamine, tyrosine, and arginine) and (4) all amino acids (Total AA: combination of EAA and NEAA). Multivariate linear regressions were used to investigate associations between protein intake from the 3-DFR and plasma amino acids. Protein intake at 12 M was divided into three forms including intake from all protein types, intake from animal source foods (ASFs)—so called “*animal-based protein—ABP*”—and intake from plant-based diets—so called “*plant-based protein—PBP*”. In multivariate models, protein intake was examined as a predictor of BCAA, EAA, NEAA and total AA concentrations. The adjusted models also included gender, type of milk feeding at 12 M (i.e., only breast milk, combined and only formula/cow’s milk) and energy intake at 12 M (kcal/day) as covariates. In addition, cross-classification was used to demonstrate the individual agreement between protein intake and plasma amino acid status. All analyses were performed using IBM SPSS version 26.0 (Armonk, NY: IBM Corp).

## 3. Results

### 3.1. Characteristics of Participants

A total of 145 healthy term infants (49% female) were included for the analysis. As shown in Table 1, the percentage of infants who did not receive other types of milk apart from breast milk alongside complementary foods was more than 50% at 9 M but dropped to about one-third at 12 M, while the use of formula or cow’s milk increased from 9 to 12 M. Mean z-scores for infant growth parameters were within the expected ranges compared with the WHO growth standard [32] at 9 and 12 M. All parents were literate and around half were college or university graduates. Notably, the mean body mass index (BMI) of fathers was in the overweight range while mean maternal BMI was normal but just 0.2 kg/m^2^ below the overweight cut-off using reference values for Asian populations [33].

### 3.2. Relative Validation at Group Level

As shown in Table 2, mean differences of all nutrient intakes between the 24-HR and 3-DFR were small and ranged between 0.6 to 7.8%, except for vitamin C intake at 12 M, which was 13.2% lower for the 24-HR compared to the 3-DFR. Mean intakes of energy and macronutrients were not different between the dietary assessment tools using paired *t*-tests. However, for micronutrients, median intakes of vitamin A at 9 M and vitamin C at 12 M from the 24-HR were significantly lower than the 3-DFR analysed by Wilcoxon signed rank tests.

**Figure 1:**
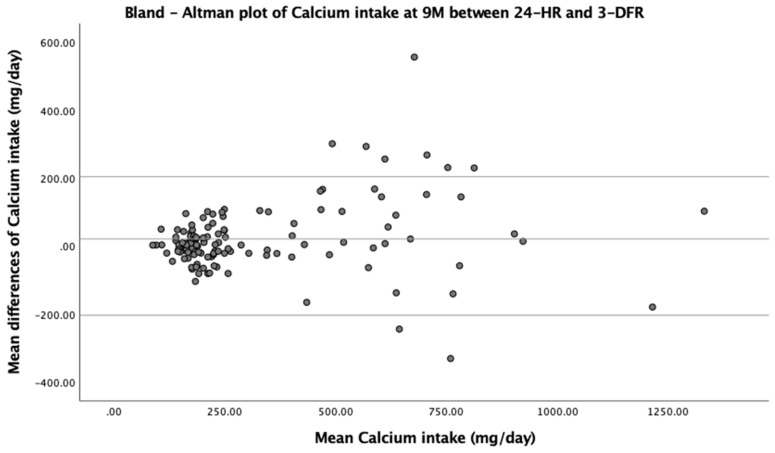

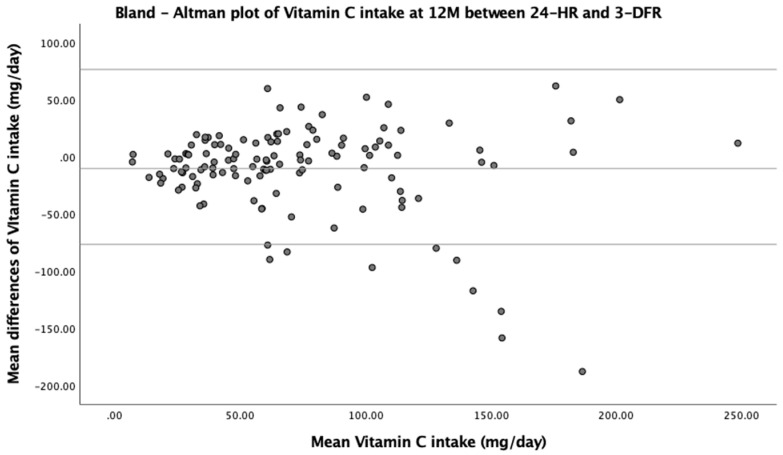
Bland–Altman plots showing the proportional biases of calcium intake at 9 M and vitamin C intake at 12 M.

A weighted kappa (Kw) statistic was used to exclude agreement by chance (Table 3). Apart from vitamin A intake at 9 M, Kw values for all nutrients were acceptable (Kw ≥ 0.2, range 0.20–0.49) with narrow 95% confidence intervals (95% CIs). As shown in Table 4, Bland–Altman analyses showed unbiased mean differences for most nutrient intakes, with only calcium intake at 9 M and vitamin C intake at 12 M showing significant biases. Figure 1 illustrates the Bland–Altman plots demonstrating the proportional biases for these two nutrients. When the mean intake was higher, the mean differences tended to increase for calcium intake at 9 M but decrease for vitamin C intake at 12 M. In other words, calcium intake at 9 M seemed to be overestimated while vitamin C intake at 12 M was likely to be underestimated when consumptions of those nutrients were higher. Additionally, when comparing the limits of agreement (LOA) for all nutrient intakes between 9 and 12 M, they were broader at 12 M particularly for macronutrient and mineral intakes. Noticeably, the LOA for vitamin A intake seemed to be widest at both 9 and 12 M compared with other nutrients.

### 3.3. Relative Validation at Individual Level

As suggested by Lombard et al. [31], cross-classification and correlation coefficient (r) were used to investigate the relative validation at individual level. As shown in Table 5, most nutrient intakes reached an acceptable level for cross-classification showing ≥50% in the same quartiles except for vitamin A intake (9 M 38.9%; 12 M 45.2%) and fat intake at 12 M (43.5%). However, when considering misclassification, none of these had more than 10% in opposite quartiles (range: 0–4.8%). In order to investigate association between the 24-HR and 3-DFR at individual level, Spearman’s correlation was used due to the skewness of micronutrient intakes. As shown in Table 5, the correlation coefficients were good to excellent (range 0.52 to 0.87) for all nutrient intakes, except for vitamin A at 9 M (r = 0.37).

### 3.4. Association of Protein Intake with Plasma Amino Acids

**Figure 2:**
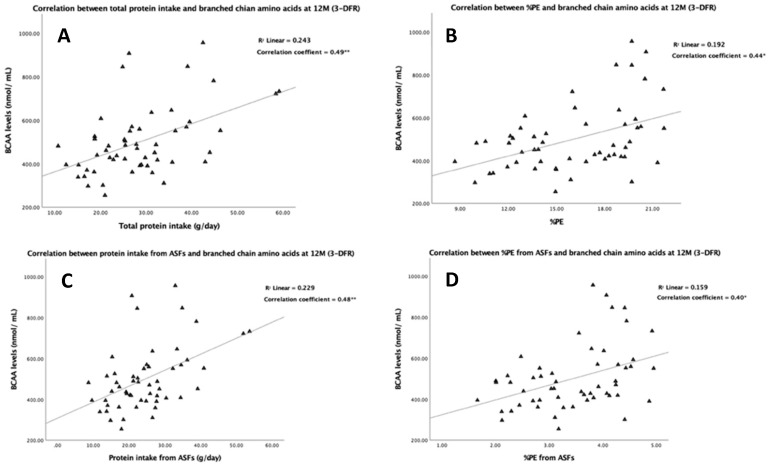

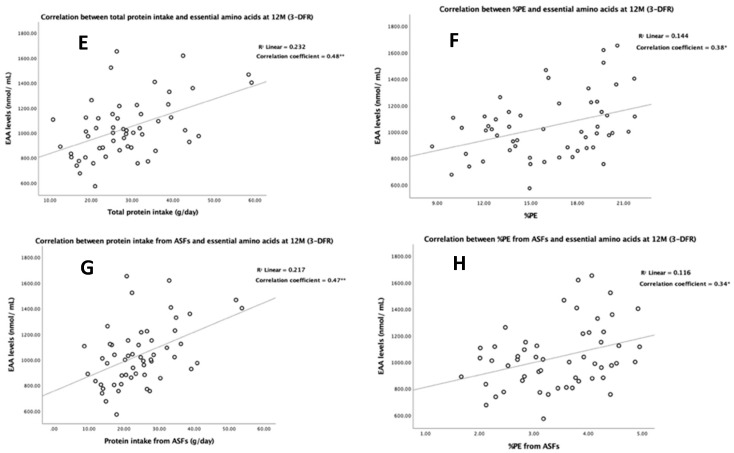
Correlations between protein intake from 3-day food records (3-DFR) and plasma amino acids at 12 M. The triangles (Δ) and circles (O) represent the associations between protein intakes and plasma levels of branched-chain amino acids (BCAA) and essential amino acids (EAA), respectively. The coefficient of determination (R^2^) and correlation coefficients (r) are shown in the right upper corner of all scatter plots. Significant *p*-values are shown as * (*p* < 0.05) and ** (*p* < 0.001). Correlations between plasma levels of BCAA and total protein intake, protein–energy percentage (%PE), protein intake from animal source foods (ASFs) and %PE from ASFs are shown in graphs A, B, C and D, respectively. Correlations between plasma levels of EAA and total protein intake, %PE, protein intake ASFs and %PE from ASFs are shown in graphs E, F, G and H, respectively.

In sub-group analyses, we compared protein intake at 12 M from the 3-DFR with plasma amino acids, as a potential biomarker for protein. Protein intakes (g/day) from all protein sources—ABP and PBP—of infants in this sub-group were normally distributed and were not different compared to protein intakes from the whole study population suggesting they were representative (data not shown). Protein intake showed significant associations with levels of BCAA, EAA and total AA before adjusting for gender, type of milk feeding and energy intake. For the adjusted models, protein intake was associated only with BCAA and EAA concentrations, with a greater effect size compared to the unadjusted models. Furthermore, only ABP intake was significantly related to BCAA and EAA levels for both regression models. According to the adjusted models, a 1 g/day increase in the consumption of ABP is associated with a 10.5 and 14.8 nmol/mL increase in plasma BCAA and EAA levels, respectively. Figure 2 shows simple scatter plots corresponding to the results in Table 6. The right column shows the associations between protein intake (g/day) from all sources or ASFs and BCAA and/or EAA levels while the left column shows the correlations between % protein–energy and BCAA and/or EAA levels. The plots show positive linear correlations between protein intake from all sources and ABP regardless of adjustment for energy intake. There was no association between intakes of PBP and plasma amino acids nor between protein intakes and NEAA. In addition, the coefficient of determinations (r^2^) indicated that around 20% of BCAA and EAA levels could be predicted by protein intake, but the proportions were slightly decreased when adjusting for energy consumption. At an individual level, the cross-classification showed poor agreement between protein intake and plasma amino acid status. Nevertheless, intake of ABP showed better agreement; a high percentage in the same quartile (42.6%) and the lowest percentage of misclassification (1.9%) was found between intake of ABP and BCAA status (Table 7).

### 3.5. Daily Variation of Energy Consumption from the 3-DFR

**Figure 3:**
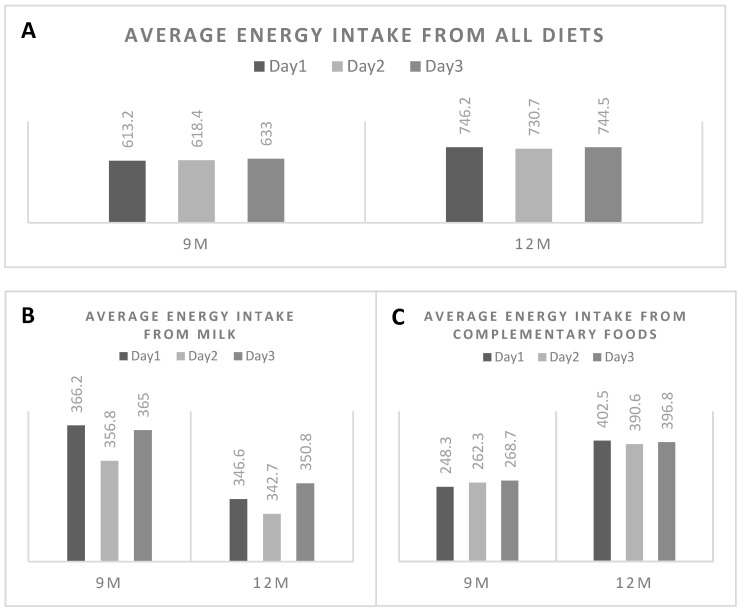
Variation in daily energy intake from 3-days food records. (**A**) shows the variation in total energy intake (kcal/day) at 9 and 12 months, while (**B**,**C**) demonstrate the variation in energy intakes from milk and complementary foods, respectively.

To investigate day-to-day variation of the reference method used in this study, we analysed the variation of daily energy consumption from the 3-DFR at 9 and 12 M (Figure 3). Small variations were observed across three days at both 9 and 12 M regardless of increasing solid food intake with lower milk consumption at 12 M. The ANOVA repeated measurements also showed non-significant differences among days 1–3 (data not shown).

## 4. Discussion

In this study, we found that the 24-HR can be used as a practical alternative to a 3-DFR for Thai infants especially during the complementary feeding period. The comparison of nutrient intakes between these methods showed favourable outcomes at both group and individual level except for some micronutrients. Vitamin A intake was the most problematic with disagreement between the two methods at both levels. Although energy and macronutrient intakes demonstrated good to excellent correlations and acceptable agreements between methods. Comparison with a protein biomarker demonstrated that protein intake, particularly ABP, predicted plasma BCAA and EAA concentrations even after adjusting for gender, type of milk feeding and energy consumption.

Few validation studies of dietary assessment tools have been conducted in infants compared to other age groups and most were in western countries or high-income settings. Recently, Beaton et al. [34] suggested that a 24-HR was a validated tool when using an estimated food record as a reference in Australian toddlers (average age 12.85M). However, only five nutrients (energy, protein, calcium, iron and added sugar) were analysed in this study. Although the results showed good agreement at group and individual levels using multiple statistical approaches including Bland–Altman analysis, a major concern was the reference method used in this study. They combined nutrient intakes from both 24-HR and 2-day food record (2-DFR) to create the reference method; so-called “24-HR + 2DFR”. These two methods are different by their nature and the chance of good agreement might be increased because the 24-HR data was also included in the reference method. From a LMIC perspective, while most of studies validated a FFQ with various types of reference methods [5,19,35,36], Hemsworth and colleagues^7^ compared the relative validity of an interactive 24-HR and one-day weighed food record in rural Malawian infants (aged 9–10 m). This study showed significant differences between mean intakes of energy and protein, while Bland–Altman analyses indicated a systematic bias from the 24-HR compared with the reference method. No individual level comparisons were made. For Malawian toddlers aged 15M, the interactive 24-HR showed good agreement at group level and acceptable agreements at individual level when compared with a weighed record [10]. However, the 24-HR significantly overestimated energy and nutrient intake in a large number of participants from the same population and may require adjustment to provide more accurate results [9].

Although the present study did not use a multiple day weighed food record as a reference, there is evidence of strong correlation and good agreement between estimated and weighed food records [11,37,38], and studies have suggested that fewer days of record are required for infants and young children [29,39]. In addition, if respondents are taught how to estimate a portion size properly, the accuracy of estimated food consumption should be improved [40]. The small day-to-day variation in energy intake observed in the estimated 3-DFR used in this study suggests it is highly reliable and justifies its use as the reference method to validate the 24-HR.

Regarding the disagreements between dietary assessment methods for micronutrient intakes, especially for vitamin A, Lombard et al. [31] linked the poor validity of vitamin A intake to irregular consumption of the good sources of vitamin A on daily basis. As we found more disagreements for vitamin A intake from many statistical tests than for other micronutrients, the dietary intakes of the outliers were re-checked. The results showed that a tablespoon of liver can make a large difference in the vitamin A intake if liver consumption was recorded on only one of the dietary assessment methods; one tablespoon of cooked pork liver and chicken liver can provide 2859 and 1344 RAE, respectively.

To our knowledge, the present study is the first to compare protein intake with plasma amino acids as a potential protein biomarker in an infant population especially during the complementary feeding period when protein consumption rapidly increases in terms of its quantity and quality [41]. Although plasma amino acid analysis is not recommended as the gold standard, this technique is more feasible and practical in infants and young children than the recommended recovery biomarker, 24-h urine nitrogen. There are several studies demonstrating an association between protein intake, especially ABP, and plasma amino acids [25,42,43,44]. Socha et al. [24] reported higher levels of BCAAs and EAAs in infants aged 6M who were randomly assigned to receive a high protein formula compared to those randomized to a low protein formula, or breast-fed. BCAA were the most determinant metabolites from metabolomic analysis in the high protein intake group [45].

The findings from our study suggested that protein intake particularly from ASFs was significantly associated with plasma BCAA and EAA levels in infants during the complementary feeding period. Although overall agreement between protein intake and plasma amino acids was poor at the individual level, the percentage of the same quartile between ABP and BCAA status was still high and nearly acceptable and showed only 1.9% of misclassification. However, further controlled studies with larger sample size are still needed to confirm the use of plasma amino acids as a protein biomarker and compare it with other dietary assessment methods.

Our study has several strengths. Firstly, the range of statistical approaches used to investigate the relative validation in this study at both group and individual level was more than in other studies [18,31]. Additionally, the overall results were consistent for each nutrient intake despite the five different approaches applied. Secondly, we also used a dietary biomarker to assess protein intake estimated by our dietary assessment method which can reduce the subjective biases from self-reported errors and improve accuracy. The high compliance is also a strength, with more than 85% of the 3-DFR and almost 100% of the 24-HR completed. Fourthly, all respondents were trained how to use their household utensils to estimate food intake. Finally, as the INMUCAL-Nutrients programme was developed using a database of local Thai foods and ingredients, the majority of foods were available in this programme and this may decrease the chance of estimation errors incurred by using less relevant food composition tables.

However, the study also has some limitations. The lack of repeated 24-HR at each time point meant that we cannot demonstrate the reliability of this tool. Although data for the 3-DR suggested the variation in energy consumption on a daily basis was small, repeated 24-HR interviews could bring other benefits particularly in assessing the intake of foods that are not regularly consumed. In addition, due to time constraints, a single field researcher had to check the 3-DFR and conduct the interviews for the 24-HR while infants were waiting for their immunization and health surveillance, and some parents did not complete the 24-HR properly during the time available. However, all were contacted later by phone to complete the 24-HR. Finally, the consumption of grains and legumes, which are better sources of PBP than cereals or vegetables, was low in our participants, and this may affect the generalizability of the non-significant association between plasma amino acids and PBP to other populations where grains and legumes are regularly consumed as part of their everyday diets.

## 5. Conclusions

Our study suggests that a 24-HR can be used as a practical alternative to a 3-DFR in this infant population during the complementary feeding period, although caution is required when assessing the intake of some micronutrients, particularly vitamin A. Protein intake, especially ABP significantly predicted levels of BCAA and EAA regardless of gender, type of milk feeding and energy consumption. However, further studies with larger sample size are needed to confirm the relationship between protein intake and plasma amino acids in infants. In order to improve accuracy of a 24-HR, a repeated interview might be needed.

## Figures and Tables

**Table 1:** Characteristics of participants (*n* = 145).

Characteristics	Results
Infant gender, n (%)	
Female	71 (49.0)
Gestational age (weeks), means ± SD	38.8 ± 1.0
Type of milk feeding, n (%)	
9 months	
- Breast milk only	79 (54.5)
- Combined	14 (9.6)
- Formula only	52 (35.9)
12 months	
- Breast milk only	46 (31.7)
- Combined	25 (17.2)
- Formula/Cow’s milk only	74 (51.0)
Anthropometric measurements, means ± SD	
9 months	
- WAZ	−0.4 ± 0.8
- WLZ	−0.1 ± 0.8
- LAZ	−0.4 ± 0.9
12 months	
- WAZ	−0.6 ± 0.9
- WLZ	−0.2 ± 0.9
- LAZ	−0.5 ± 0.9
Parental BMI (kg/m^2^), means ± SD	
- Maternal BMI	22.8 ± 4.0
- Paternal BMI	24.7 ± 3.6
Maternal education, n (%)	
- Lower than bachelor’s degree	76 (52.4)
- Bachelor’s degree or higher	69 (47.6)
Paternal education, n (%)	
- Lower than bachelor’s degree	87 (60.0)
- Bachelor’s degree or higher	58 (40.0)
Family income per month * (Thai Bath), n (%)	
- <10,000	11 (7.6)
- 10,000–29,999	65 (44.8)
- 30,000–49,999	51 (35.2)
- ≥50,000	18 (12.4)
Completeness of dietary data, n (%)	
24-h dietary recall	
- At 9 months	142 (97.9)
- At 12 months	144 (99.3)
3-day food record	
- At 9 months	129 (89.0)
- At 12 months	125 (86.2)

* minimum wage in Chiang Mai was 320 baht per day during period of data collection (from National Wage Committee’s notification on Minimum Wage rate No. 9, 2018–2019). WAZ—Weight-for-age z-score; WLZ—Weight-for-length z-score; LAZ—Length-for-age z-score; BMI—Body mass index; SD—Standard deviation.

**Table 2:** Paired *t*-test/Wilcoxon signed rank test and mean difference of daily nutrient intakes compared between 24-h dietary recalls (24-HR) and 3-day food records (3-DFR).

	9 Months				12 Months			
Energy and Macronutrients	24-HR means ± SD	3-DFR means ± SD	Mean difference (%)	*p* *	24-HR means ± SD	3-DFR means ± SD	Mean difference (%)	*p* *
Energy (kcal)	624.5 ± 193.9	630.0 ± 191.4	−5.6 (0.9)	0.60	725.5 ± 236.9	747.0 ± 222.5	−21.6 (2.9)	0.14
CHO (g)	78.1 ± 25.0	79.7 ± 24.5	−1.7 (2.1)	0.28	86.7 ± 31.1	88.9 ± 28.5	−2.2 (2.5)	0.28
Fat (g)	25.6 ± 8.7	25.6 ± 8.7	0.04 (0.2)	0.94	29.1 ± 12.0	30.4 ± 10.7	−1.3 (4.3)	0.14
Protein (g)	19.8 ± 8.4	20.2 ± 7.9	−0.4 (2.0)	0.41	28.4 ± 10.5	29.3 ± 10.1	−0.8 (2.7)	0.21
% Caloric distribution - CHO - Fat - Protein	50.4 ± 6.1 37.1 ± 5.7 12.4 ± 2.8	51.0 ± 5.3 36.3 ± 5.4 12.7 ± 2.6	−0.6 (1.2) 0.8 (2.2) −0.2 (1.6)	0.28 0.12 0.36	48.3 ± 8.0 35.8 ± 6.5 15.9 ± 3.7	47.8 ± 6.6 36.5 ± 5.5 15.7 ± 3.0	0.5 (1.0) −0.7 (1.9) 0.1 (0.6)	0.48 0.27 0.57
Micronutrients	24-HR Median (IQR)	3-DFR Median (IQR)	Mean difference (%)	*p* **	24-HR Median (IQR)	3-DFR Median (IQR)	Mean difference (%)	*p* **
Calcium (mg)	234.0 (170.7, 539.2)	231.0 (172.3, 415.4)	20.4 (6.4)	0.07	406.9 (209.3, 617.0)	371.1 (194.7, 662.5)	5.1 (1.2)	0.78
Phosphorous (mg)	261.2 (174.5, 461.6)	251.3 (195.4, 377.6)	3.5 (1.1)	0.91	415.8 (254.7, 583.6)	406.4 (261.6, 627.9)	−8.8 (2.0)	0.43
Iron (mg)	3.2 (1.9, 8.3)	3.1 (2.2, 7.0)	0.05 (1.0)	0.83	5.1 (2.7, 8.9)	4.4 (2.9, 9.0)	−0.3 (4.8)	0.36
Zinc (mg)	2.3 (1.5, 5.0)	2.3 (1.6, 4.3)	0.1 (3.2)	0.30	3.6 (2.2, 5.1)	3.3 (2.2, 5.3)	−0.1 (2.5)	0.97
Vitamin A (RAE)	563.8 (371.8, 1298.3)	1105.0 (478.9, 1736.6)	−47.4 (3.7)	0.03	474.3 (352.4, 781.3)	610.8 (381.9, 1229.0)	−47.4 (4.0)	0.05
Vitamin B1 (mg)	0.3 (0.2, 0.6)	0.3 (0.2, 0.5)	−0.01 (2.5)	0.11	0.4 (0.3, 0.6)	0.4 (0.3, 0.7)	0.004 (0.8)	0.70
Vitamin B2 (mg)	0.5 (0.3, 1.2)	0.5 (0.4, 1.1)	0.02 (2.9)	0.74	0.9 (0.5, 1.3)	0.9 (0.5, 1.3)	0.003 (0.3)	0.68
Vitamin C (mg)	60.5 (32.5, 95.4)	59.5 (41.6, 96.0)	−5.8 (7.8)	0.10	58.8 (35.7, 92.8)	64.2 (40.9, 64.2)	−10.3 (13.2)	0.04

* *p*—*p*-values from paired *t*-tests; ** *p*—*p*-values from Wilcoxon signed Rank tests. RAE—Retinol activity equivalent; CHO—Carbohydrate; IQR—Interquartile range.

**Table 3:** Agreements of energy and nutrient intakes between 24-HR and 3-DFR.

Nutrients	9 Months		12 Months	
	Kw	95% CI	Kw	95% CI
Energy (kcal)	0.326	0.323, 0.329	0.301	0.298, 0.304
CHO (g)	0.281	0.279, 0.284	0.337	0.334, 0.339
Fat (g)	0.281	0.279, 0.284	0.195	0.192, 0.198
Protein (g)	0.352	0.350, 0.355	0.275	0.272, 0.277
Calcium (mg)	0.352	0.350, 0.355	0.434	0.431, 0.437
Phosphorous (mg)	0.432	0.429, 0.435	0.372	0.369, 0.375
Iron (mg)	0.361	0.358, 0.364	0.372	0.369, 0.375
Zinc (mg)	0.485	0.483, 0.488	0.443	0.440, 0.445
Vitamin A (RAE)	0.148	0.146, 0.151	0.213	0.210, 0.215
Vitamin B1 (mg)	0.352	0.349, 0.355	0.336	0.334, 0.339
Vitamin B2 (mg)	0.361	0.358, 0.364	0.301	0.298, 0.304
Vitamin C (mg)	0.317	0.314, 0.320	0.275	0.272, 0.277

Kw—Weighted kappa; CI—Confident interval; RAE—Retinol activity equivalent.

**Table 4:** Bland–Altman analyses.

Nutrients	9 Months			12 Months		
	Mean Differences	LOA ± 1.96 SD	Slope of Biases (*p*-Value)	Mean Differences	LOA ± 1.96 SD	Slope of Biases (*p*-Value)
Energy (kcal)	−5.6	±233.7	−0.52 (0.60)	−21.6	±318.9	−1.48 (0.14)
CHO (g)	−1.7	±33.8	−1.09 (0.28)	−2.2	±44.3	−1.08 (0.28)
Fat (g)	0.04	±12.5	0.07 (0.94)	−1.3	±19.6	−1.47 (0.14)
Protein (g)	−0.4	±10.7	−0.83 (0.41)	−0.8	±14.5	−1.26 (0.21)
Calcium (mg)	20.4	±203.9	2.20 (0.03)	5.1	±289.3	0.39 (0.70)
Phosphorous (mg)	3.5	±177.5	0.43 (0.67)	−8.8	±263.4	−0.73 (0.47)
Iron (mg)	0.05	±3.5	0.30 (0.76)	−0.3	±4.7	−1.46 (0.15)
Zinc (mg)	0.1	±1.6	1.75 (0.08)	−0.1	±3.6	−0.54 (0.59)
Vitamin A (RAE)	−47.4	±3466.3	−1.01 (0.32)	−47.4	±4063.2	−0.25 (0.80)
Vitamin B1 (mg)	−0.01	±0.2	−0.93 (0.36)	0.004	±0.4	0.24 (0.81)
Vitamin B2 (mg)	0.02	±0.6	0.88 (0.38)	0.003	±0.7	0.10 (0.92)
Vitamin C (mg)	−5.8	±66.5	−1.91 (0.06)	−10.3	±76.7	−2.92 (0.004)

LOA—Limits of agreement.

**Table 5:** Cross-classification and Spearman’s correlations of nutrient intakes compared between 24-HR and 3-DFR.

Nutrients	9 Months			12 Months		
	% Same Quartiles	% Opposite Quartiles	Correlation Coefficient *	% Same Quartiles	% Opposite Quartiles	Correlation Coefficient *
Energy (kcal)	54.8	0	0.79	53.2	1.6	0.72
CHO (g)	50.8	0	0.74	56.5	2.4	0.65
Fat (g)	50.8	0.8	0.75	43.5	4.8	0.59
Protein (g)	57.1	0.8	0.75	50.8	1.6	0.53
Calcium (mg)	57.1	0	0.85	65.3	0	0.86
Phosphorous (mg)	64.3	0	0.81	59.7	0	0.81
Iron (mg)	57.9	0	0.82	59.7	0.8	0.85
Zinc (mg)	69.0	0	0.87	66.1	0.8	0.80
Vitamin A (RAE)	38.9	4.8	0.37	45.2	4.8	0.52
Vitamin B1 (mg)	57.1	0	0.81	56.5	0	0.75
Vitamin B2 (mg)	57.9	0	0.78	53.2	0	0.79
Vitamin C (mg)	54.0	0.8	0.74	50.8	0	0.74

* *p*-value < 0.001 for all nutrients.

**Table 6:** Multivariate regression analysis predicting plasma amino acids by protein intake at 12 M.

Protein Intake (g/day)	BCAA Levels		EAA Levels		NEAA Levels		Total AA Levels	
	Crude *β*	Adjusted ^1^ *β*	Crude *β*	Adjusted ^1^ *β*	Crude *β*	Adjusted ^1^ *β*	Crude *β*	Adjusted ^1^ *β*
All sources	7.4 **	11.2 *	10.9 **	15.8 *	4.2	3.8	15.0 *	19.6
ABP	7.8 **	10.5 *	11.5 **	14.8 *	4.1	3.7	15.6 *	18.5
PBP	31.3 *	28.4	48.1 *	39.1	25.8	6.5	73.8 *	45.6

^1^ Controlled variable in the adjusted model included gender, type of milk feeding and total energy intake * *p* < 0.05: ** *p* < 0.001. ABP—Animal-based protein; PBP—Plant-based protein; BCAA—Branched-chain amino acids; EAA—Essential amino acids; NEAA—Non-essential amino acids; AA—Amino acids.

**Table 7:** Cross-classification between protein intake at 12 M and plasma amino acids levels.

Protein Intake (g/d)	BCAA		EAA		NEAA		Total AA	
	% Same Quartile	% Opposite Quartile	% Same Quartile	% Opposite Quartile	% Same Quartile	% Opposite Quartile	% Same Quartile	% Opposite Quartile
All sources	44.4	1.9	38.9	3.7	27.8	7.4	24.1	5.6
ABP	42.6	1.9	37.0	3.7	29.6	9.3	29.6	7.4
PBP	33.3	7.4	29.6	9.3	27.8	11.1	38.9	13.0

ABP—Animal-based protein; PBP—Plant-based protein; BCAA—Branched-chain amino acids; EAA—Essential amino acids; NEAA—Non-essential amino acids; AA—Amino acids.
